# Long-Range Signaling in MutS and MSH Homologs via Switching of Dynamic Communication Pathways

**DOI:** 10.1371/journal.pcbi.1005159

**Published:** 2016-10-21

**Authors:** Beibei Wang, Joshua Francis, Monika Sharma, Sean M. Law, Alexander V. Predeus, Michael Feig

**Affiliations:** Department of Biochemistry & Molecular Biology, Michigan State University, East Lansing, MI, United States; Koç University, TURKEY

## Abstract

Allostery is conformation regulation by propagating a signal from one site to another distal site. This study focuses on the long-range communication in DNA mismatch repair proteins MutS and its homologs where intramolecular signaling has to travel over 70 Å to couple lesion detection to ATPase activity and eventual downstream repair. Using dynamic network analysis based on extensive molecular dynamics simulations, multiple preserved communication pathways were identified that would allow such long-range signaling. The pathways appear to depend on the nucleotides bound to the ATPase domain as well as the type of DNA substrate consistent with previously proposed functional cycles of mismatch recognition and repair initiation by MutS and homologs. A mechanism is proposed where pathways are switched without major conformational rearrangements allowing for efficient long-range signaling and allostery.

## Author Summary

We are proposing a new model for how long-range allosteric communication may be accomplished via switching of pre-existing pathway as a result of only minor structural perturbations. The systems studied here are the bacterial mismatch repair enzyme MutS and its eukaryotic homologs where we identified strong communication pathways connecting distant functional domains. The functionally-related exchange of nucleotides in a distant ATPase domain appears to be able to switch between those pathways providing a new paradigm for how long-range allostery may be accomplished in large biomolecular assemblies.

## Introduction

Allostery is a fundamental part of many if not most biological processes. It is classically defined as the induced regulation at one site by an event at another distal site. Venerable models for allostery, such as the MWC (Monod-Wyman-Changeux) [1] and KNF (Koshland-Nemethy-Filmer) [2] models emphasize a mostly static picture of induced conformational changes. The MWC model proposes coupled conformational changes via a population shift while the KNF model highlights the induced-fit of a binding of a ligand via common communication routes. A broader view of allostery [3–6] emphasizes communication pathways via protein motions but without requiring actual conformational changes. The idea of this model is that relatively minor perturbations may shift communication between multiple pre-existing pathways. Such a mechanism has been demonstrated by nuclear magnetic resonance (NMR) experiments for the binding of cyclic-adenosine monophosphate (cAMP) to the dimeric catabolite activator protein (CAP) [7] as well as for allosteric regulation in Pin1[8]. Recent work based on Markov state models that integrate energetics and kinetics has added further nuances to the discussion by emphasizing both conformational and kinetic selection as the main mechanism of allostery in signaling proteins protein kinase A [9] and NtrC [10]. The idea of kinetic selection is consistent with a pathway selection mechanism without significant conformational changes. Recent reviews have attempted to integrate the different ideas into a unified view [11, 12] with the main question being to what degree conformational dynamics plays a role. Likely, the degree of dynamics will depend on a given system and the economics of achieving allosteric signaling within the thermodynamic and functional constraints in the biological environment. One particular question that is central to this work is how long-range allostery can be achieved in very large systems where larger conformational changes and global selection mechanisms that are conceptually straightforward in smaller proteins could be more challenging to realize.

It is difficult to obtain detailed insight into allostery from experiments, especially for larger and more complex systems, because NMR spectroscopy is generally limited to small and soluble proteins that can be easily labeled and expressed in large quantities. On the other hand, crystallography is not well-suited for studying allosteric effects due to its inherent dynamic nature.

Computational approaches such as statistical coupling analysis (SCA) [13], normal mode analysis (NMA) [14, 15], dynamical network analysis [16], and Markov state model analysis based on extensive molecular dynamics simulations [9, 10] offer complementary means for exploring allosteric mechanisms in biological systems. SCA, a bioinformatics-based method, obtains allosteric information by identifying coevolving residues from multiple sequence alignments, while NMA, a structure-based approach, suggests induced movements from a few robust low-frequency normal modes. Allosteric pathways obtained from these two methods would be encoded in the sequence and/or structure, but sensitivity to minor perturbations with this type of analysis is lacking. Dynamical network analysis [16] is based on molecular dynamics (MD) simulations and has been used to identify synchronous and/or asynchronous correlated residue motions in order to describe possible allosteric communication pathways. Examples of where this approach has been applied successfully to probe allosteric coupling include a tRNA-protein complex [16], the M2 muscarinic receptor [17], and cysteinyl tRNA synthetase [18]. Here, we used dynamical network analysis to develop a paradigm for allostery in very large multi-subunit complexes based on long-range signal propagating pathways in the MutS component of the methyl-directed DNA mismatch repair (MMR) system.

MMR is responsible for correcting errors that escape immediate proofreading during DNA replication and the mechanism is widely conserved from prokaryotic to eukaryotic organisms. MMR alone can increase the accuracy of DNA replication by 20–400 fold [19]. While several components, such as MutS, MutL, MutH, nuclease, and polymerase, are needed to work together to complete DNA repair [20], MutS is responsible for the initial recognition of DNA lesions, in particular mismatches and insertions or deletions (IDLs). MutS is a homodimer, but, structurally and functionally, it acts as a heterodimer because only one subunit (termed the ‘A’ chain in this paper) directly contacts the lesion sites [21]. MutS homologs (MSH) in eukaryotes are heterodimers with differing substrate specificities. MutSα (MSH2-MSH6) preferentially recognizes base pair mismatches and single base IDLs [22], whereas MutSβ (MSH2-MSH3) has a higher affinity and specificity for small DNA loops composed of 2–13 bases [23].

**Fig 1:**
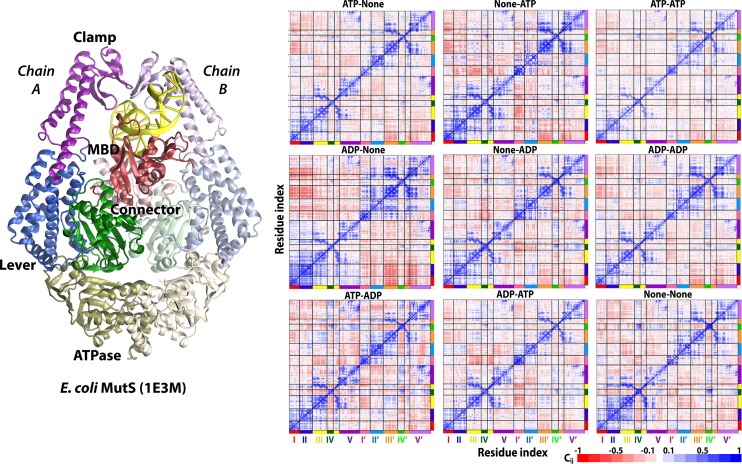
*(left)* Structure of E. coli MutS with major domains defined and colored as follows: mismatch binding domain (MBD, 1–125), red; connector domain (126–286), green; lever domain (287–419, 538–567), blue; clamp domain (420–537), purple; nucleotide-binding ATPase domain (568–800), olive; *(right)* dynamical cross-correlation matrices for Cα atoms for *E*. *coli* MutS simulated systems as a function of nucleotides bound to ATPase domains. Magnitudes of calculated cross-correlations *c*_*ij*_ are indicated by the color bar. See also S1 Fig.

The crystal structures of prokaryotic MutS and its eukaryotic homologs, complexed with mismatched DNA heteroduplexes, feature a similar overall Θ shape [22, 24–26]. Each subunit of MutS and MSH is comprised of five distinct domains (see Fig 1): the mismatch-binding domain (MBD, domain I), the connector domain (domain II), the lever domain (domain III), the clamp domain (domain IV), and the nucleotide binding domain (ATPase, domain V) [24]. The MBD and clamp domains interact with the bound DNA directly. The MBD contains a conserved, mismatch-identifying Phe-X-Glu motif, forming specific interactions with mismatches. The phenylalanine forms an aromatic ring stack on the 3′ side of the mismatched base [24, 25] although there is also evidence for base flipping of the mismatched or neighboring base during the mismatch recognition process [27, 28]. MSHβ, which specializes in the recognition of longer insertions/deletions, lacks this motif. The lever and connector domains connect the MBD and clamp domains to the ATPase domain. The ATPase domain is a conserved domain in the ABC (ATP binding cassette) superfamily. Biochemical studies have provided evidence that ATPase activities are coupled with DNA scanning, mismatch recognition, and repair initiation [29–31]. The different functional states are assumed to involve different conformations of MutS. The major states are MutS without DNA with open clamps, MutS scanning DNA in search of a mismatch with the clamps closed, MutS bound to a mismatch in the tightly DNA-bound conformation seen in crystallography, and a sliding clamp configuration where MutS is able to move away from the mismatch without scanning or complete dissociation from the DNA [32]. Based on the biochemical data, nucleotide binding and exchange to the ATPase domain appear to be key allosteric effectors coupled to DNA mismatch recognition that at least in part trigger changes between those functional states. This implies allosteric coupling between the mismatch binding site and the ATPase site over a distance of 70 Å [33] is essential for the biological function of MutS. Mismatched binding promotes exchange from ADP to ATP based on kinetic measurements of ATP hydrolysis [30, 34, 35] and results in asymmetric activity of the two ATPase domains [30], whereas the sliding clamp state is supposed to be formed in ATP binding states [36–38].

Previous studies have examined MutS and eukaryotic analogs via molecular dynamics simulations [27, 32, 39–43], but many mechanistic questions remain. Here, we subjected previously generated simulations of MutS, MutSα (MSH2-MSH6) and MutSβ (MSH2-MSH3) to dynamical network analysis to elucidate allosteric communication pathways between the structural domains in MutS and MSHs. In particular, we addressed the questions of how intra-molecular signaling could be accomplished over very long distances via protein dynamics and how small perturbations could affect the signal propagation. Previous work has suggested coupling between the MBD and ATPase domains, but mechanistic details and in particular the role of exchanging NTPs still remain largely unclear [43, 44]. The dynamic network analysis applied here allowed us to probe for pathways connecting the domains in contact with the DNA and the ATPase domain. Furthermore, by comparing pathways in simulations with different nucleotides bound to MutS and different DNA substrates bound to MutSα and MutSβ we were able to develop hypotheses for how communication along those pathways may be shifted during the functional cycle of MutS and its homologs.

## Results

### Structural Variations in MutS

A number of very similar MutS and MSH crystal structures are available with different nucleotide bound states and different mismatches or IDLs. The structural variations that can be discerned primarily focus on the MBD, ATPase and clamp domains and involve mostly local side-chain displacements rather than larger conformational changes of the main chain. For example, the MutS crystal structures 1E3M (with a single ADP) [24] and 1W7A (with bound ATP) [33] differ by only 0.35 Å in the Cα coordinates after superposition. MD simulations paint a similar picture. In previous work from our group, MD simulations of MutS with all possible combinations of nucleotides bound to the ATPase dimer did not reveal large conformational changes of the overall MutS structure based on RMSD and clustering analysis [27]. A similar conclusion was found for *Thermus aquaticus* MutS in a recent study [44], although different nucleotides bound to the ATPase domain were not examined. Taken together, this information has suggested that allosteric communication in this system likely takes place via subtle changes in local dynamics to achieve signaling in MutS rather than via conformational selection or induced conformational changes [3, 44].

### Direct Domain Correlations in MutS

Average dynamical cross-correlation matrices (DCCM) were calculated from the MD simulations. Fig 1 compares the DCCMs between MutS simulations with different nucleotides. A comparison of the DCCMs after 50, 100, 150, and 200 ns generally shows little change after 50 ns (S1 Fig). This suggests that the correlations based on 200 ns trajectories are well converged consistent with a previous study [45]. In all cases, we found strong local correlation within domains but also weaker coupling between distant parts of the complex (Fig 1). Overall, different nucleotide bound states resulted in similar coupling patterns, but differences as a function of different nucleotide bound states can be discerned. For example, the positive MBD(I)-connector(II)^A^ coupling is strongest in ADP-None, while the strongest positive MBD(I)-connector(II)^B^ coupling is observed in None-ADP. Also in the case of ADP-None, the MBD and connector domains of subunit A are strongly negatively coupled with the lever and clamp domains of subunit B. The two clamp domains are positively coupled in cases of ATP-ADP and None-None, which are stronger than the others. The positive coupling between the two ATPase domains is strongest in ATP-ADP. Similar direct correlations between MutS domains have also been observed in other work based on MD simulations of *Thermus aquaticus* MutS [44]. However, while a direct correlation analysis suggests coupling, it does not provide complete information about the pathway(s) along which allosteric communication take place and it discounts the possibility of asynchronous communication via stochastic steps that would introduce a variable time delay between signal input and output along a given communication pathway.

### Dynamic Communication Pathways in MutS

Next, we turned to dynamical network analysis to allow for a more dynamic model of allostery where direct correlations between distant sites are not required. In this approach, pathways connecting residue pairs along the shortest path with the highest pairwise local correlations based on the converged DCCMs from 200 ns MD sampling are determined. We focused our analysis on the functionally most relevant signal propagation between the MBD, ATPase, and clamp domains using specific key residues as anchor points (S2 Table). A first set of pathways was determined between MutS-F36, the key residue in direct contact at the mismatch site, and MutS-K620, the key residue involved in binding the phosphate tails of NTPs in the ATPase domain. A second set of pathways was focused on the communication between the two ATPase domains connecting MutS-K620 in the A and B chains and a third set of pathways was constructed from MutS-K620 to MutS-N497, which is the contact point of the clamp domain with the DNA opposite the mismatch site in the B subunit of MutS.

**Fig 2:**
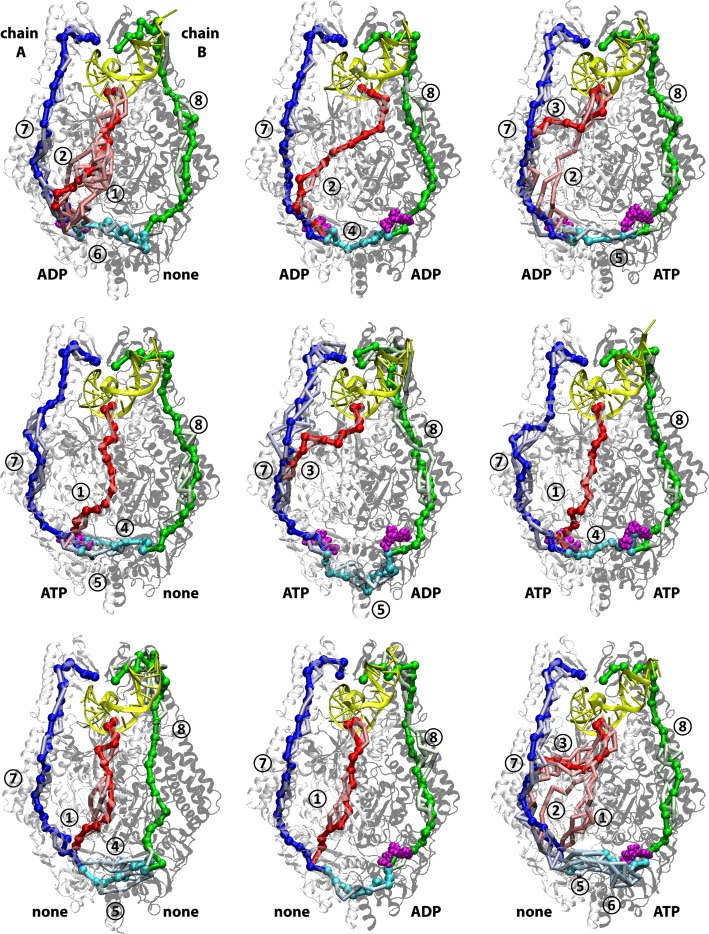
MBD-ATPase (red), ATPase-clamp (blue/green), and ATPase-ATPase (cyan) allosteric communication pathways from MD-based correlation analysis mapped on the MutS structure as a function of different nucleotide-bound states. Optimal pathways are shown in saturated colors, alternate suboptimal pathways are shown in lighter colors. Major pathways are indicated with circled numbers (see Tables 1–3 for more details). See also S2 Fig.

Mapping of the resulting pathways onto the MutS structure is shown in Fig 2. The computational analysis suggests multiple major pathways that vary as a function of the nucleotides bound to the ATPase domain. Within each major pathway, there are ensembles of similarly optimal minor pathways. The variability in the pathways was greatest within a given structural domain, where strong coupling between many residues allowed for many alternate, equivalent routes. However, connections between domains were limited to certain key residue pairs (S3 Table) that presented bottlenecks in the respective pathways. When employing network analysis to group strongly coupled residues into communities (S2 Fig), these communication bottlenecks appear as critical inter-community edges that are hypothesized to correspond to switching points between major pathways when perturbed.

**Table 1:** Properties of optimal paths in MutS between mismatch binding site (F36) and ATP binding site (K620) in chain A as a function of nucleotides bound in the ATPase domains.

	①			②			③		
	N	W	min	N	W	min	N	W	min
**ADP-None**	18	264	0.56	**21**	**266**	**0.73**			
**ADP-ADP**				18	327	0.70			
**ADP-ATP**				16	329	0.68	23	330	0.71
**ATP-None**	17	329	0.52						
**ATP-ADP**							**22**	**240**	**0.73**
**ATP-ATP**	16	293	0.56						
**None-ADP**	16	308	0.53						
**None-ATP**	17	256	0.58	19	257	0.72	23	258	0.83
**None-None**	17	285	0.55						
**E169P: ADP-None**				18	281	0.70	19	287	0.70
**L240D: ADP-None**							19	285	0.62
**Q626A: ATP-ADP**				18	297	0.65			
**L558R-A: ATP-ADP**				17	289	0.52	23	285	0.73
**L558R-B: ATP-ADP**							22	210	0.80

Pathways: ① F36-MBD-connector (150–280)-ATPase-K620; ② F36-MBD-connector-lever (300–330)-ATPase-K620; ③ F36-MBD-lever/helix (540–560)-ATPase-K620.

N: number of hops; W: overall weight calculated as $W=-100\;\sum_{k}\log\left|c_{ij}^{k}\right|$; min: minimum pairwise correlation c_ij_ along path.

**Table 2:** Properties of optimal paths in MutS between ATP binding sites (K620) in chains A and B and the clamp domain (N497) as a function of nucleotides bound in the ATPase domains.

	⑦			⑧		
	N	W	min	N	W	min
**ADP-None**	27	392	0.72	**28**	**209**	**0.86**
**ADP-ADP**	27	376	0.70	27	319	0.81
**ADP-ATP**	28	324	0.80	28	382	0.76
**ATP-None**	27	389	0.73	26	327	0.76
**ATP-ADP**	**28**	**277**	**0.79**	28	325	0.81
**ATP-ATP**	27	331	0.67	29	325	0.83
**None-ADP**	28	325	0.79	28	288	0.83
**None-ATP**	27	309	0.82	26	240	0.80
**None-None**	27	293	0.80	26	347	0.59
**Q626A: ATP-ADP**	28	359	0.79	28	314	0.83
**L558R-A: ATP-ADP**	28	306	0.75	30	286	0.83
**L558R-B: ATP-ADP**	27	243	0.79	28	260	0.86

Pathways: ⑦ A:K620-A:lever-A:clamp-A:N497; **⑧** B:K620-B:lever-B:clamp-B:N497.

*N*, *W*, and *min* were calculated as in Table 1.

**Table 3:** Properties of optimal paths in MutS between ATP binding sites (K620) in chains A and B as a function of nucleotides bound in the ATPase domains.

	④			⑤			⑥		
	N	W	min	N	W	min	N	W	min
**ADP-None**							12	202	0.70
**ADP-ADP**	10	257	0.55						
**ADP-ATP**				9	207	0.57			
**ATP-None**	10	245	0.54	11	251	0.61			
**ATP-ADP**				**13**	**154**	**0.76**			
**ATP-ATP**	9	217	0.52						
**None-ADP**							10	214	0.64
**None-ATP**				11	251	0.61	14	248	0.74
**None-None**	12	258	0.65	9	254	0.54			

Pathways: ④ A:K620-B:690-700-B:K620; ⑤ A:K620-A:690-700-B:K620; ⑥ A:K620-A:670-680-B:770-780-B:K620.

*N*, *W*, and *min* were calculated as in Table 1.

Tables 1–3 quantify the features of the optimal pathways in terms of the number of steps (hops) required to traverse a path from the beginning to the end, a weight reflecting the degree of correlation along the optimal path, and the minimum pairwise correlation for any residue pair along the path. This analysis was carried out for each of the three sets of pathways as a function of different nucleotides bound in the ATPase domains. The algorithm employed here is designed to always find an optimal path connecting two given residues. In order to identify paths that are functionally relevant we focused on paths that stand out by having significantly lower weights than other paths while also requiring that the minimum correlation along the path was at least 0.7. Our assumption is that even if a path has an overall low weight, it would not be an effective route of communication if it contained one or more links with poorly coupled residues.

#### MBD-ATPase pathways

Three major types of MBD-ATPase pathways were identified (see Fig 2 and Table 1): The first path (①) would go from the MBD through the connector and then directly to the ATPase domain; the second path (②) would involve helix α10 of the lever domain (residues 300–330) as an intermediate between the connector and the ATPase domains; the third path (③) would connect from the MBD directly to helix α13 of the lever domain, largely skipping the connector, before reaching the ATPase domain. Communication along path ① appears to be inefficient, because some steps along the path have a low pairwise correlation (below 0.52–0.58).

**Fig 3:**
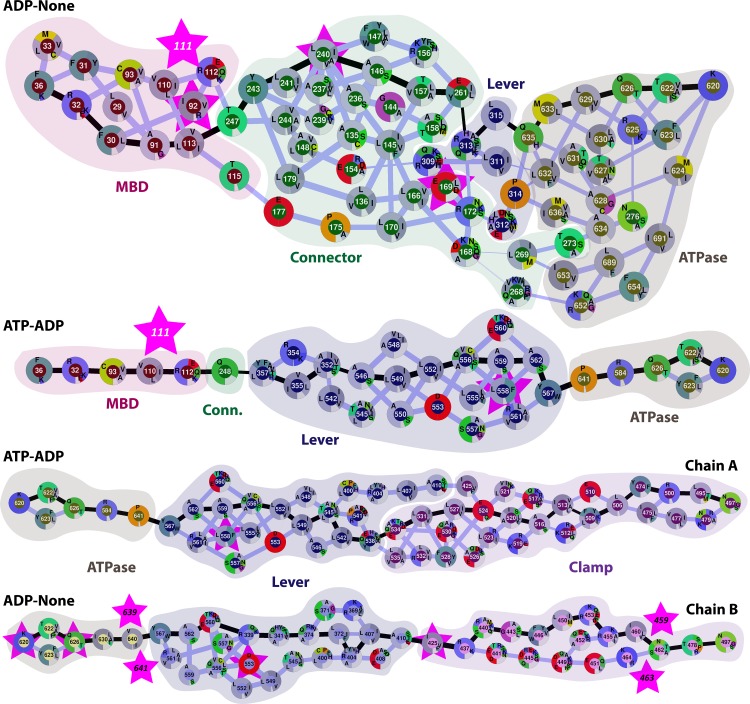
Network diagrams of proposed communication pathways between the MBD and ATPase domains along paths ② (ADP-None), and ③ (ATP-ADP) (top) and between the ATPase and clamp domains along paths ⑦ (ATP-ADP) and ⑧ (ADP-None) (bottom). Nodes correspond to residues with amino acid conservation across bacterial and eukaryotic homologs indicated as a pie chart for each residue. Edges are drawn based on optimal (black) and suboptimal (grey) allosteric paths. The thickness of lines corresponds to the pairwise direct correlations extracted from the MD simulations. Cancer-associated mutations in MSH6 (top three paths) and MSH2 (bottom path, chain B of MutS) mapped onto MutS residues and highlighted with pink stars. See also S3 Fig, S4 Fig and S5 Fig.

Focusing then on states where either ADP or ATP is bound in the A (mismatch-bound) domain of the MutS dimer, we find two paths with significantly lower weights: path ② for ADP-None and path ③ for the ATP-ADP state. These paths are visualized in more detail in Fig 3.

Communication along path ② appears to involve many alternate routes, rather than a single well-defined path. A broad ensemble of possible paths is a consequence of considering just slightly suboptimal paths (see Methods) as found also in related work from the Amaro group [46]. Such an ensemble of alternate routes would provide robustness with respect to mutations and/or structural perturbations. Most residues along the paths are highly conserved across bacterial and eukaryotic homologs of MutS. Some residues, especially those near domain junctions such as 112, 158, 168, 261, 268, 309, 312, and 313 are variable, but changes remain mostly within the same type of amino acid (charged/polar residues). This would suggest coupling mostly via electrostatic interactions.

**Fig 4:**
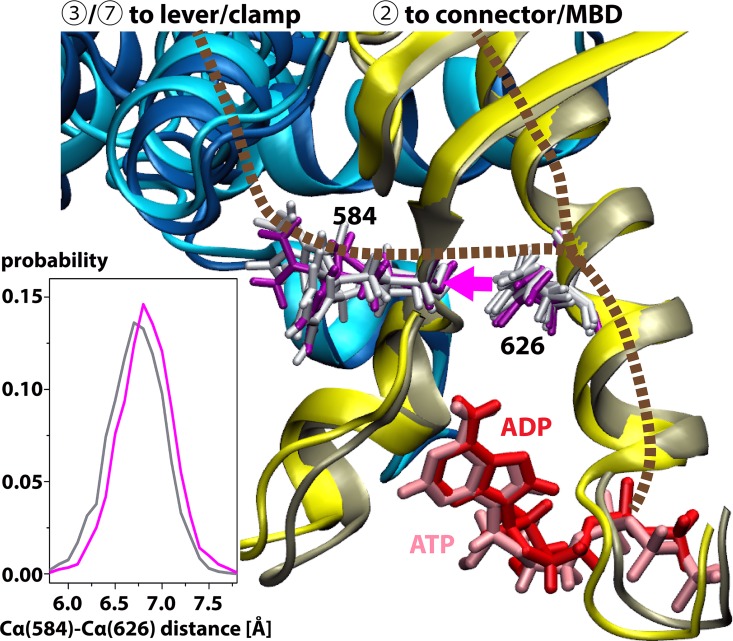
Close-up comparison of parts of the ATPase and lever domains in the ADP-None and ATP-ADP states near the nucleotide binding site in subunit A. The structures shown are representative conformations obtained from clustering of superimposed simulation snapshots. The ADP-None state is shown in darker colors (red, tan, blue) while the ATP-ADP state is shown in lighter colors (pink, yellow, cyan). Communication paths from the nucleotide binding site to the lever or the connector are indicated schematically in brown. Residue 626 is the branching point from which communication either proceeds to the connector or switches to the lever via residue 584. For both residues, side chain conformations obtained from clustering of simulation trajectories are shown in purple (ADP-None) and white (ATP-ADP), respectively. The inset on the lower left shows the distribution of Cα-Cα distances between residues 584 and 626 from the MD simulations of the ADP-None (magenta) and ATP-ADP (grey) states.

It is readily apparent that path ③ has only very little overlap with path ② except for a few residues near the start (F36) and end (K620) points. This suggests that simply exchanging ADP for ATP in the A site may be sufficient to switch between entirely different pathways. In order to understand the structural basis in more detail we compared the detailed conformations of the pathway residues in the simulations with the ADP-None and ATP-ADP states. As shown in Fig 4, the presence of ATP leads to a shift in the ATPase domain in subunit A around the nucleotide binding site as ATP extends deeper into the binding pocket than ADP. One consequence of that is that the distance between residues Q626 and R584 becomes slightly shorter when ATP is bound compared to the ADP-None state. While the shift in distance of around 0.15 Å may seem insignificant, we calculated the interaction energies between residues 626 and 584 from snapshots with ADP-None and ATP-ADP. We find that the interaction becomes more favorable in the presence of ATP by 0.5–1.0 kcal/mol by comparing electrostatic and van der Waals energies for just the two residues. The stronger contact could explain how the dynamic correlation is enhanced from 0.73 for the ADP-None state to 0.89 for the ATP-ADP state. We rationalize that the shorter distance between Q626 and R584 in the ATP-ADP state favors communication along path ③ and instead of path ②.

**Fig 5:**
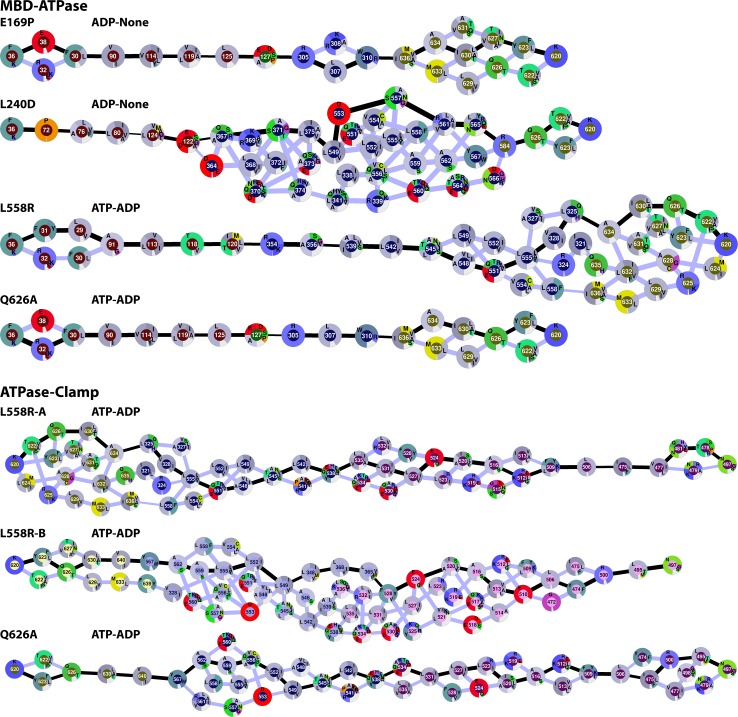
Network diagrams of optimal communication pathways between the MBD and ATPase domains in MutS mutants E169P/ADP-None, L240D/ADP-None, Q626A/ATP-ADP, and L558R/ATP-ADP and between the ATPase and clamp domains for Q626A/ATP-ADP and L558R/ATP-ADP as in Fig 3.

Q626, which is almost perfectly conserved along with R625 [47], is therefore be a prime target for mutational studies. Specifically we hypothesize that smaller residues would disrupt the ability of MutS to switch pathways and carry out its function. To test this idea computationally, we ran an additional simulation of a Q626A mutant in the ATP-ADP state. The resulting optimal path is shown in Fig 5 with quantitative analysis results given in Table 1. With this mutant, optimal communication is only seen along path ② instead of path ③, suggesting that, indeed, this residue is critical in allowing the proposed pathway switching from ② to ③ when ADP is exchanged for ATP.

A large number of mutations in the human MutS homolog MSH6-MSH2 are known to be associated with cancer phenotypes [48]. Most mutations cause frameshifts or early termination of translation which is expected to lead to a complete loss of structure and function of MSH6 or MSH2. More interesting are the smaller number of point mutations that are believed to cause defective mismatch repair (S4 Table and S5 Table) [47]. We found four cancer-associated MSH6 point mutations extracted from the UMD [49] and Insight/LOVD [50] databases that map onto residues involved in pathway ② (using a previously published alignment [24]). Two mutations involve highly connected central residues (169 and 240) and three are near the domain junctions (92, 111, 169) where effective communication would be critical in our proposed pathways. Our main hypothesis is that these mutations disrupt the proposed communication pathways, but without further studies we cannot rule out that the identified mutations do not act primarily by compromising MutS structure or MutS-MutL interactions.

Four out of 16 non-frameshift mutations in MSH6 that map onto MutS coincide with one of the 97 pathway residues shown in Fig 2 with one additional mutation found next to two pathway residues. Given a total number of 800 residues for MutS, we would expect to find only two out of 16 residues to coincide with the pathway residues in a random distribution. This suggests that our findings are statistically significant although one or two of the mapped mutations could be fortuitous.

To further test whether mutations hypothesized to impact communication between the MBD and ATPase domains we examined the E169P and L240D mutants in the presence of ADP-None. As shown in Fig 5 and Table 1, both mutants weaken communication from the MBD to the ATPase domain via path ② in favor of path ③, especially for the L240D mutant, consistent with our idea that communication along path ② is necessary for initial signaling of a mismatch-bound state.

Path ② is a plausible communication route that could be taken in the presence of ADP in the A site to communicate mismatch recognition to the ATPase site. Mismatch recognition is experimentally known to be followed by exchange of ADP for ATP. Our analysis suggests that once such an exchange takes place, the communication along path ② is lost and instead communication along the lever (path ③) would be preferred. In generating path ③, we required that a connection is made between the MBD and ATPase domains. However, path ③ largely overlaps with communication to the clamp (see below) suggesting that exchange from ADP to ATP may in fact switch communication between the MBD and the ATPase to communication between ATPase and the clamp domain and/or changes in the lever and core domain. We hypothesize that the latter could also place MutS into a MutL-binding conformation, the next functional step in the MutS mismatch repair cycle.

#### ATPase-clamp pathways

We identified two major pathways connecting the ATPase domain with the clamp, one along chain A, the other along chain B. All of the paths would connect through the lever along the long kinked helix (helices α20 and α21) as suggested by the crystal structures [24, 25]. We observe again an ensemble of paths variations when considering slightly suboptimal paths (see Fig 2). Based on the quantitative analysis shown in Table 2, the optimal paths in all but one case (None-None, path ⑧) do not have steps with low pairwise correlations, and, for many cases, the minimum pairwise correlation is remarkably high, above 0.8, along the entire path with almost 30 steps. Path ⑦ along the A chain for ATP-ADP and path ⑧ along the B chain for ADP-None have significantly lower weights than the other paths. These two paths are shown in more detail in Fig 3. As mentioned above, path ⑦ for ATP-ADP overlaps significantly with path ③ with the branching points at residues 546 and 542 where communication coming from the ATPase domain could either proceed to the clamp or to the MBD domain according to our analysis. Since the coupling between 542–538 and 546–542 (about 0.94) is stronger than 546/542-352 (about 0.78) we would hypothesize that communication from the ATPase domain may be primarily directed at the clamp along this path when ATP-ADP are present. The optimal path through the B chain with ADP-None appears to follow a slightly different path than in chain A, which may be a result of the structural asymmetry of the MutS complex. Cancer-associated mutations in MSH6 and MSH2 were again mapped onto the pathways. One MSH6 mutation maps onto path ⑦ at the central, highly connected residue 558. In addition, there are a significant number of MSH2 mutations that map onto different parts of path ⑧. One mutation corresponds to a central residue (553), while other mutations are located at or next to residues involved in critical domain junctions (next to 640; 425; next to 460, 462, and 464) of the proposed paths. Mutations of neighboring residues may affect the contacts of residues on the paths and thereby change their dynamics. We would thus hypothesize that these mutations could disrupt the allosteric communication along path ⑧. For MSH2, we mapped four out of 35 mutations onto one of 47 pathway residues with four additional mutations located immediately adjacent to pathway residues which compares with two out of 35 (corresponding to the 47/800 ratio) mutations in a random match onto pathway residues.

To test the proposed importance of 558, we carried out simulations of L558R mutants in either chain A or B. Results are shown in Tables 1 and 2 with optimal pathways depicted in Fig 5. This mutation has a small effect on the communication between the MBD and the ATPase domain, since path ③ clearly remains the dominant communication path. However, communication from the ATPase domain to the clamp domain is weakened in chain A. Residue 558 is still involved in the pathway but assumes a less central role (Fig 5). In chain B, residues 558 appears to be less important for communication between the ATPase domain and communication may actually be strengthened with the L558R mutation in chain B. Finally, we also analyzed the ATPase-clamp communication along chain A for the Q626A mutant. Again, the 626–584 interaction is lost and an alternative path is taken with a higher weight compared to the wild type MutS (see Table 2) suggesting that Q626 is indeed a key residue for communication to and from the ATPase domain.

The ADP-None combination (ADP in chain A, no nucleotide in chain B) is believed to be the mismatch scanning configuration of MutS [30]. We would speculate that a strong communication along path ⑧ could be necessary to maintain tight interactions with the DNA when probing for mismatches. N497 of chain B is positioned opposite the mismatch recognition site and would hold the DNA in place when scanning. Based on our analysis, we propose that exchange of ADP for ATP following mismatch recognition may weaken the interaction along chain B while strengthening communication along chain A. However, the implications for how exactly the clamp domains would be reconfigured as a consequence of communication along the proposed paths are unclear since we did not actually observe significant clamp dynamics in the simulations underlying this study.

#### ATPase^A^-ATPase^B^ pathways

**Fig 6:**
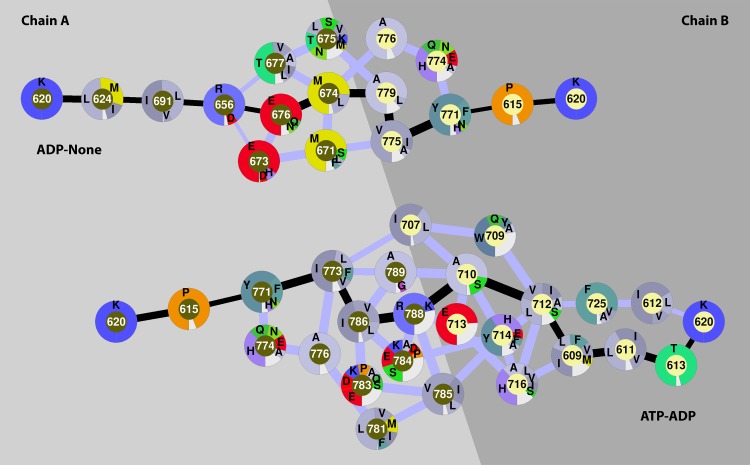
Network diagrams of proposed communication pathways between the two ATPase domains along paths ⑤ (ATP-ADP) and ⑥ (ADP-None) as in Fig 3. See also S6 Fig and S7 Fig.

Finally, we examined the shorter-range communication between the two ATPase domains (S3 Fig for structural details of the ATPase domains). There is experimental evidence that such communication is important to step from an initial mismatch scanning ADP-None state to ATP-None and then ATP-ATP/ATP-ADP states [51]. The ATPase^A^-ATPase^B^ pathways obtained from our computational analysis can be roughly classified into three types (Fig 2 and Table 3): The first, path ④, goes directly from the Walker A motif of chain A (residues 614–622) to the D-loop (residues 696–700) and then the Walker B (residues 688–694) and Walker A motifs of chain B (S6 Fig). In the second path ⑤, the Walker A motif of chain A is connected to the Walker B motif, then to the ABC signature loop (residues 663–676) still within chain A before connecting to the B chain via the Helix-turn-helix (HTH) dimerization domain (residues 766–800) (Fig 6). Finally, in path ⑥, the Walker A motif connects to the HTH motif in chain A, and from there to residues 707–716 in chain B before connecting to the Walker A motif (Fig 6). The quantitative data in Table 3 suggests that the strongest communication would follow path ⑤ for the ATP-ADP combination. Path ⑥, the apparently preferred route for the ADP-None and None-ADP/ATP states, may also be efficient since the correlation coefficients are high. Path ④ has a low minimum correlation along the path for all but one combination (None-None) suggesting that this path may not be as effective as the other two paths. While path ⑥, seen for the mismatch scanning ADP-None state, does not involve either the signature loop or the Walker B motif, we hypothesize that exchange of ADP for ATP could switch the communication to either path ⑤ or ④ which would engage the signature loop in path ⑤ and the Walker B motif of chain B in path ④. The ABC signature loop has been proposed previously to be able to modulate the conformational changes associated with ATP binding/hydrolysis [52]. Furthermore, it was found that mutations on the ABC signature loop allow mismatch recognition but prevent sliding clamp state [53]. The combination of the experimental data with our analysis suggests a model where exchange of ADP for ATP and binding of ADP or binding of ATP followed by hydrolysis in the B site upon mismatch recognition to reach the ATP-ADP state establishes strong coupling between the ATPase domains via the signature loop. As discussed above, coupling between the ATPase domains and between the ATPase and clamp domains is assumed to be important for transitioning from the mismatch recognition state to the sliding clamp.

### Proposed model for long-range allosteric communication mechanism

**Fig 7:**
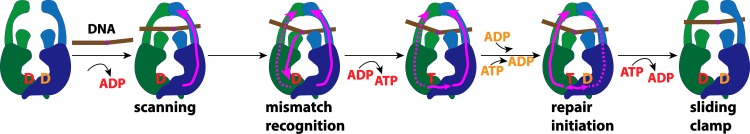
A proposed mechanism of how switched communication paths with alternate nucleotide-bound states facilitates mismatch repair initiation.

The overall premise of this study is the development of a dynamic allostery model for MutS since structural and previous simulation data suggest little conformational change as a function of nucleotides bound to the ATPase domains. Such a model implies the presence of communication paths between key structural elements (MBD, ATPase, and clamp domains) and the main result of this work is the identification of such paths in a nucleotide-dependent manner. Integrating previous biochemical data with such a dynamic allosteric model allowed us to arrive at the mechanism depicted in Fig 7 and described in more detail in the following:

In the absence of DNA, the ADP-ADP state is presumed to be dominant [54]. The ADP-ADP state dissociates directly from DNA, while the binding of DNA induces the dissociation of one ADP molecule, more likely to be the one in B subunit. Therefore, the ADP-None state is presumed to be the mismatch scanning state [34, 55]. Crystal structures of MutS were also obtained mostly in the ADP-None state [56–59]. Our results also support this idea. The MBD^A^ strongly couples with the connector and ATPase domains in the ADP-None state via the proposed pathway ② that consists of a broad ensemble of individual paths with a few bottlenecks at domain boundaries. This communication is then proposed to result in exchange of ADP for ATP in the ATPase^A^ domain upon mismatch recognition [34, 35]. In our model, the presence of ATP in the A site would abolish the communication between the MBD and ATPase^A^ domains because an optimal or suboptimal path via the connector domain is either absent altogether (ATP-ADP) or present with less favorable weights or low minimum pairwise correlations (ATP-none, ATP-ATP in path ①) that suggest inefficient coupling. At the same time, the ATPase^A^ -ATPase^B^ communication would engage the Walker B motif of chain B when ATP is present in the A site. We further hypothesize that ADP or ATP binding to the B site would follow, leading to the ATP-ADP state. Since the lifetime of the ATP-None state is believed to be short [54] this would occur quickly. Once the ATP-ADP state is reached, our model suggests that the ATPase^A^ domain connects to the lever instead of the connector. Because the connector primarily connects with the MBD domain while the lever domain provides a route to the clamp domain, we hypothesize that in the ATP-ADP state communication from the ATPase^A^ domain would be switched from the MBD domain to the clamp. At the same time, strong coupling between the ATPase^A^ -ATPase^B^ domains via the signature loop could disrupt the strong ATPase^B^-clamp connection present in the ADP-None state and allow release of MutS from the mismatch site.

The mechanism above postulates communication routes within the context of a dynamic allosteric mechanism that could be tested further experimentally, e.g. via mutations of pathway residues. Based on the dynamics sampled in the underlying simulations we are able to propose a structural basis for how pathways are switched in the presence of different nucleotides, however, the model is still lacking a clear mechanism for how ADP would be exchanged for ATP following mismatch recognition, for how the clamp domains would respond to signaling resulting from nucleotide exchange as proposed here, and what role MutL binding plays in this process. We speculate that the altered correlated dynamics induces subtle shifts in the overall conformational landscape which would favor ADP-ATP exchange and lead to clamp opening. To address this idea in more detail, significant additional simulations are required to probe the DNA binding process and clamp dynamics leading to the sliding clamp conformation in excess of the scope of the present work. Such a model could also conceptually integrate recent conformational landscape-based ideas of allostery with the communication-focused analysis presented here into a complete model for allostery in a large, complex system such as MutS where simpler concepts of conformational selection or induced-fit may not be able to adequately describe the allosteric mechanism.

### Allosteric communication pathways shift with different DNA substrates

MutS can recognize a broad range of lesions, mismatches and IDLs, but MSHs have differentiated substrate specificities. MutSα (MSH2-MSH6) primarily recognizes mismatches and single base IDLs, whereas MutSβ (MSH2-MSH3) recognizes DNA loops composed of 2–13 bases. Based on previous simulations of MutSα and MutSβ with native and swapped substrates and no DNA at all [60], we also analyzed how different DNA substrates would shift the signaling pathways identified via our computational analysis.

**Fig 8:**
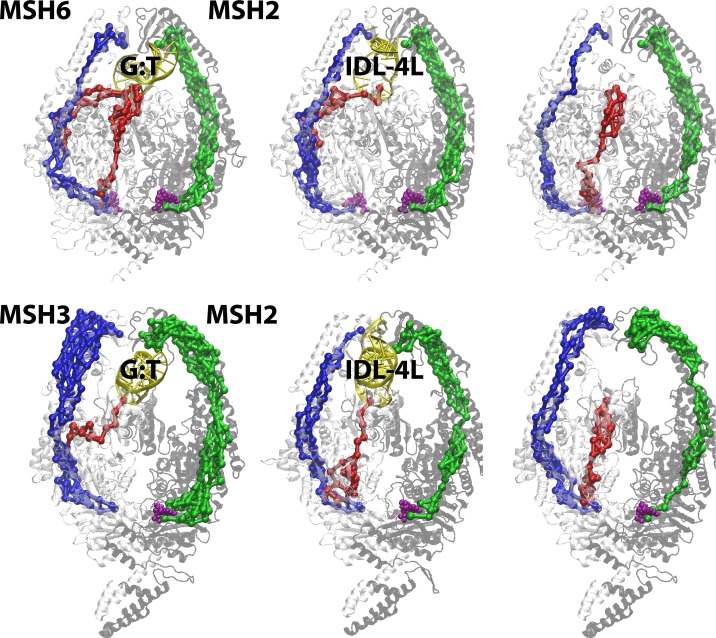
MBD-ATPase (red) and ATPase-clamp (blue/green) allosteric communication pathways from MD-based correlation analysis mapped on the MSH6/2 and MSH3/2 structures as a function of different DNA substrates. Optimal pathways are shown in saturated colors, alternate suboptimal pathways are shown in lighter colors. See also S8 Fig and S9 Fig.

In the MSH complexes we identified pathways analogous to paths ①, ②, and ③ in MutS (see Fig 8 and details in S8 Fig and S9 Fig) suggesting that the proposed communication pathways may be preserved in the eukaryotic homologs. There appears to be strong communication from the MBD through the connector domain when MutSα and MutSβ are bound to their native substrates (a G:T mismatch and a four-nucleotide insertion loop (IDL-4L), respectively). However, swapping the substrate would abolish that path in favor of coupling along the lever domain. Again, cancer-associated mutations in MSH6 and MSH2 map onto the paths, some at critical edges connecting different domains (see S7 Fig and S8 Fig). Interestingly, communication between the MBD and ATPase domain of MSH3/MSH6 through the connector would also be present in the absence of DNA. These findings expand our allosteric model where effective communication between the MBD and ATPase^A^ domains (and subsequent initiation of repair) would depend on both the nature of the DNA substrate and the nucleotides bound in the ATPase domains.

## Discussion

Long-range signaling and allostery is a key mechanistic component of many large biomolecular complexes. Here, we present a detailed analysis of *E*.*coli* MutS and MSHs where several long-range signaling steps are essential for initiating DNA repair following mismatch recognition. Using dynamic network analysis based on extensive molecular dynamics simulations we developed a model consisting of a number of communication pathways that depend on strong local pairwise residue dynamical coupling where signaling would be expected to progress stochastically along those paths. In this model, different combinations of ATPase-bound nucleotides would result in switching between different pathways to implement the functional cycle of MutS without significant conformational rearrangements. A signaling mechanism based on pre-existing pathways that are switched on or off by different nucleotides and/or different DNA substrates is consistent with previous crystallographic and simulation studies that show surprisingly little structural variations in mismatch-bound MutS and homologs. The benefit of such a mechanism could be energetic economy, especially when considering the very long range over which the pathways appear to operate. Experimental validation of the hypotheses presented here could involve mutations of key residues, but it will also be interesting to see whether similar mechanisms are at play in other large enzymes. However, further computational studies will also be necessary to develop a more complete mechanistic understanding of how exactly signaling along the proposed pathways would promote and depend on nucleotide exchange and how it would lead to sliding clamp formation and complex formation with MutL.

## Materials and Methods

### Simulation details

MD simulations of the *E*.*coli* MutS protein bound to a G:T mismatch DNA (PDB ID: 1W7A) [33] were previously performed [27]. Each ATPase site may have three states: ATP, ADP or no nucleotide. All combinations of the three states in either of the two ATPase domains were simulated. They are denoted as ATP-None, None-ATP, ATP-ATP, ADP-None, None-ADP, ADP-ADP, ATP-ADP, ADP-ATP and None-None (S1 Table). In this notation, the first nucleotide is present in the ATPase site of the mismatch-binding moiety (subunit ‘A’) and the second one in the ATPase site of the non-mismatch-binding moiety (subunit ‘B’). Additional new simulations were carried out for five mutants of the *E*. *coli* MutS system to test the mechanistic hypotheses developed in this study: E169P (ADP-None), L240D (ADP-None), and Q626A (ATP-ADP) in chain A as well as L558R in either chain A or B (ATP-ADP). These simulations were simulated using the same protocol as the previous simulations of the wild-type systems (see below).

MD simulations of human MutSα and MutSβ were started from the crystal structure 2O8B [22] and 3THX [26] (MutSα/G:T and MutSβ/IDL-4L) [61]. In MutSα and MutSβ structures, MSH6 and MSH3 are the mismatch-bound moieties (equivalent to the A subunit in MutS), while MSH2 interacts with the DNA non-specifically (equivalent to subunit B in MutS). Additional simulations were carried out for *apo* structures, where the DNA heteroduplex was removed (MutSα/Apo and MutSβ/Apo), and for MutSα and MutSβ where the respective substrates were swapped (MutSα/IDL-4L and MutSβ/G:T) [61].

In total, 15 previous simulations and five new simulations (S1 Table), each for at least 200 ns, were analyzed. All of the simulations were carried out with NAMD 2.8 [62] using the CHARMM27 force field [63], the latest force field available at the time those simulations were initiated. All systems were solvated in explicit solvent using the TIP3P water model and sodium counterions to neutralize the systems. Simulations were carried out under periodic boundary conditions with the particle-mesh Ewald method [64] to calculate electrostatic interactions at constant temperature (300K) and constant pressure (1 atm) using a Langevin thermostat and barostat. The fully solvated systems consisted of about 165,000 atoms for the MutS systems and about 600,000 atoms for the larger MutSα and MutSβ systems. All of the systems remained overall stable with RMSD values of 3–5 Å for Cα atoms with respect to the initial experimental structures. Additional details of the system setup and simulation results are described in our previous papers [27, 61]. VMD [65] was used to visualize and analyze simulations and generate structural figures.

### Analysis of allosteric communications

Allosteric networks within the proteins were identified using the *NetworkView* plugin of VMD [16, 66]. The dynamic networks were constructed using data from our molecular dynamics simulations of the protein-DNA complexes described above, each sampled every 1 ps. For each molecular system, a network graph was constructed with two nodes for each nucleotide (at N1/N9 and Pα/P), while protein residues were represented with a single node at the Cα position. All of the conformations from a given trajectory were pooled to calculate the local-contact matrix. A contact between two nodes (excluding neighboring nodes) was defined as within a distance of 4.5 Å for more than 75% of MD trajectories. The resulting contact matrix was then weighed by the correlation values of the two end nodes in the dynamical network as *w_ij_* = −log(|*C_ij_*|), where *C*_*ij*_ are the elements of the correlation matrix calculated as $C_{ij}=\left\langle \Delta r_{i}\cdot \Delta r_{j}\right\rangle /{\left\langle \Delta r_{i}^{2}\right\rangle }^{1/2}{\left\langle \Delta r_{j}^{2}\right\rangle }^{1/2}$. The correlation matrices, also called dynamic cross-correlation matrices (DCCM), were calculated using the carma software [67]. The length of a path is the sum of the edge weights between the consecutive nodes along this path. And the optimal (shortest) paths between two nodes in the network were obtained by the Floyd-Warshall algorithm [68]. The number of optimal paths that cross one edge is termed as betweenness of the edge. Suboptimal paths within a certain limit (offset) between the two nodes were also determined in addition to the optimal path. The number of suboptimal paths shows the path degeneracy. Communities were calculated based on the dynamical network by the Girvan–Newman algorithm [69]. The nodes in one community are more compactly interconnected than other nodes.

All pathways were determined between residues in the MBD (located within 10 Å of the mismatch site) and residues in the ATPase domain (located within 10 Å of a bound nucleotide) or between residues in the clamp domain (within 10 Å of DNA) and residues in the ATPase domain (within 10 Å of a bound nucleotide). The residue pairs with the shortest optimal path were finally selected as representative residues (S2 Table). Suboptimal paths between specific residue pairs were calculated with edge length offsets of 3, 5 and 10 for the MBD-ATPase, ATPase-ATPase, and ATPase-clamp interactions, respectively.

## Supporting Information

S1 Table Summary of the MD simulations used in this study. (DOCX)

S2 Table Related to Fig 2, Fig 3, Fig 5, Fig 7 and Tables 1–3. Selected residues used as anchor points in network analysis in MutS and eukaryotic homologs.
(DOCX)

S3 Table Related to Fig 2, Fig 3, and Tables 1–3. Critical inter-domain edges in MutS with major edges shown in bold
(DOCX)

S4 Table Related to Fig 3. Cancer-associated non-frameshift/non-mistranslation mutations in MSH2
(DOCX)

S5 Table Related to Fig 3. Cancer-associated non-frameshift/non-mistranslation mutations in MSH6
(DOCX)

S1 Fig Related to Fig 1. Dynamical cross-correlation matrices for Ca atoms for E. coli MutS simulated systems as a functions of nucleotides bound to ATPase domains using 50, 100, 150, and 200 ns of the simulation trajectories (from left to right).
(TIF)

S2 Fig Related to Fig 2. Communities of the MutS protein in all MutS systems (left: A monomer, right: B monomer). Different communities were colored differently.
(TIF)

S3 Fig Related to Fig 3. MBD-ATPase^A^ pathways for additional NTP states.
(TIF)

S4 Fig Related to Fig 3. ATPase^A^–clamp pathways for additional NTP states.
(TIF)

S5 Fig Related to Fig 3. ATPase^B^–clamp pathways for additional NTP states.
(TIF)

S6 Fig Related to Fig 5. ATPase^A^ -ATPase^B^ pathways for additional NTP states.
(TIF)

S7 Fig Related to Fig 5. The conserved motifs in NBD domain.
(TIF)

S8 Fig Related to Fig 7. MBD-ATPase pathways in MutSα and MutSβ with native DNA substrates as in Fig 3.
(TIF)

S9 Fig Related to Fig 7. ATPase-clamp pathways in MutSα and MutSβ with native DNA substrates as in Fig 3. Cancer-associated mutations are highlighted with stars.
(TIF)
