# Supplementary material for: Long-Range Signaling in MutS and MSH Homologs via Switching of Dynamic Communication Pathways
Source: PLoS Comput Biol. 2016 Oct 21;12(10):e1005159. doi: 10.1371/journal.pcbi.1005159 (PMC5074593; doi:10.1371/journal.pcbi.1005159)
Supplement: S1 Table — (DOCX) [file pcbi.1005159.s001.docx]

**Table S1.** Summary of the MD simulations used in this study.

| System | DNA | ATPase sites | | Simulation time | Abbreviations |
| --- | --- | --- | --- | --- | --- |
|  |  | A | B |  |  |
| MutS  (*E. coli*) | G:T | ATP | - | 200ns | ATP-None |
|  | G:T | - | ATP | 200ns | None-ATP |
|  | G:T | ATP | ATP | 200ns | ATP-ATP |
|  | G:T | ADP | - | 200ns | ADP-None |
|  | G:T | - | ADP | 200ns | None-ADP |
|  | G:T | ADP | ADP | 200ns | ADP-ADP |
|  | G:T | ATP | ADP | 200ns | ATP-ADP |
|  | G:T | ADP | ATP | 200ns | ADP-ATP |
|  | G:T | - | - | 200ns | None-None |
| MutS mutants | G:T | ADP | - | 200ns | E169P:A |
|  | G:T | ADP | - | 200ns | L240D:A |
|  | G:T | ATP | ADP | 200ns | Q626A:A |
|  | G:T | ATP | ADP | 200ns | L558R:A |
|  | G:T | ATP | ADP | 200ns | L558R:B |
| MSH2/MSH6  (human MutSα) | G:T | ADP | ADP | 220ns | MutSα/G:T |
|  | - | ADP | ADP | 220ns | MutSα/Apo |
|  | IDL-4L | ADP | ADP | 220ns | MutSα/IDL-4L |
| MSH2/MSH3  (human MutSβ) | IDL-4L | ADP | - | 220ns | MutSβ/IDL-4L |
|  | - | ADP | - | 220ns | MutSβ/Apo |
|  | G:T | ADP | - | 220ns | MutSβ/G:T |

A site: ATPase domain of mismatch-binding subunit (MSH3/MSH6 in MutSα and MutSβ); B site: ATPase domain of non-specific DNA binding subunit (MSH2 in MutSα and MutSβ)
