# Supplementary material for: Long-Range Signaling in MutS and MSH Homologs via Switching of Dynamic Communication Pathways
Source: PLoS Comput Biol. 2016 Oct 21;12(10):e1005159. doi: 10.1371/journal.pcbi.1005159 (PMC5074593; doi:10.1371/journal.pcbi.1005159)
Supplement: S2 Table — Selected residues used as anchor points in network analysis in MutS and eukaryotic homologs. (DOCX) [file pcbi.1005159.s002.docx]

**Table S2, Related to Figures 2-3, Figure 5, Figure 7, and Tables 1-3.** Selected residues used as anchor points in network analysis in MutS and eukaryotic homologs.

| Domain | MutS | MutSα-MSH2 | MutSα-MSH6 | MutSβ-MSH2 | MutSβ-MSH3 |
| --- | --- | --- | --- | --- | --- |
| MBD | F36 |  | F432 |  | P277 |
| Clamp | N497 | R524 | G1002 | R524 | I747 |
| NBD | K620 | G674 | Y1287 | G674 | S895 |
