# Supplementary material for: Long-Range Signaling in MutS and MSH Homologs via Switching of Dynamic Communication Pathways
Source: PLoS Comput Biol. 2016 Oct 21;12(10):e1005159. doi: 10.1371/journal.pcbi.1005159 (PMC5074593; doi:10.1371/journal.pcbi.1005159)
Supplement: S3 Table — Critical inter-domain edges in MutS with major edges shown in bold (DOCX) [file pcbi.1005159.s003.docx]

**Table S3, Related to Figures 2-3 and Tables 1-3.** Critical inter-domain edges in MutS with major edges shown in bold

| system | | Critical edges |
| --- | --- | --- |
| MBD-connector | A | V111/**R112/I113-T247**/Q248  **T115-E177**, G117-A176  P116/G117-N131  T118-I150 |
| Connector-ATPase | A | **N276/L277-R625**  I268/M269-R652/I653  T273-A634 |
| Connector-Lever | A | **R172-H312/M313**  R128/Q129-K308, Q129-R305  R154-R309  E261-M313  Q248-L357 |
| Lever-ATPase | A | **P314/V315-Y635**/I636  W310/L311-I636  L311/P314-L632  **R324-I636/G637**  I328-S638  **Y567-V640/P641**  **L558-S638**  L565/Y567-R584 |
|  | B | **Y567-V640/P641**  **L558-S638** |
| Lever-Clamp | A | **A410-I425**  **Y538/I539-E534/V535**  L365-F528 |
|  | B | **A410-I425**  **H538/L539-L534/V535**  L365-Y528 |
| ATPase-ATPase | A | **N616**/M617(A) – **T699**/**F670**/M671(B)  R697(A) – N616(B)  M671/M674/**T675**/A678(A) – V775/A776/**A779**/G780/V781(B)  A779(A) – M674/T675(B)  **T699**(A) – **Y771**(B)  L773(A) – L707(B)  L707(A) – L773(B)  V785/R788/A789(A) – A710/E713/N714(B)  H728(A) – T699/S700 (B) |
