# Supplementary material for: Long-Range Signaling in MutS and MSH Homologs via Switching of Dynamic Communication Pathways
Source: PLoS Comput Biol. 2016 Oct 21;12(10):e1005159. doi: 10.1371/journal.pcbi.1005159 (PMC5074593; doi:10.1371/journal.pcbi.1005159)
Supplement: S4 Table — Cancer-associated non-frameshift/non-mistranslation mutations in MSH2 (DOCX) [file pcbi.1005159.s004.docx]

**Table S4, Related to Figure 3.** Cancer-associated non-frameshift/non-mistranslation mutations in MSH2

| **Source** | ***h*MSH2** | ***E. coli* MutS** |
| --- | --- | --- |
| UMD | L173R | F155 |
| UMD | C199R | L178 |
| Insight | Q264H | P231 |
| Insight | L330P | L295 |
| Insight | G338E | G303 |
| UMD/Insight | L341P | M306 |
| UMD/Insight | P349H | P314 |
| UMD | I356K | L321 |
| UMD/Insight | R359S | R324 |
| Insight | V470G | I425 |
| Insight | G504R | G459 |
| UMD | I511N | L463 |
| UMD/Insight | R524H | Q476 |
| UMD | S554C | P504 |
| UMD | G587R | P537 |
| Insight | D603G | D553 |
| UMD | F608S | L558 |
| Insight | P622L | P570 |
| UMD/Insight | A636P | *N/A* |
| UMD | H639Y | H585 |
| Insight | P652H | A598 |
| UMD/Insight | G669V | G614 |
| UMD/Insight | G674D | G619 |
| Insight | K675E | K620 |
| UMD | R680P | R625 |
| UMD | Q681P | Q626 |
| Insight | G683E | A628 |
| UMD/Insight | G692W | G637 |
| Insight | F694S | Y639 |
| UMD/Insight | P696L | P641 |
| UMD/Insight | C697R | A642 |
| UMD | S723F | S668 |
| UMD | D748Y | D693 |
| UMD/Insight | E749K | E694 |
| Insight | G751R | G696 |
| Insight | A763P | A708 |
