# Supplementary material for: Long-Range Signaling in MutS and MSH Homologs via Switching of Dynamic Communication Pathways
Source: PLoS Comput Biol. 2016 Oct 21;12(10):e1005159. doi: 10.1371/journal.pcbi.1005159 (PMC5074593; doi:10.1371/journal.pcbi.1005159)
Supplement: S5 Table — Cancer-associated non-frameshift/non-mistranslation mutations in MSH6 (DOCX) [file pcbi.1005159.s005.docx]

**Table S5, Related to Figure 3.** Cancer-associated non-frameshift/non-mistranslation mutations in MSH6

| **Source** | ***h*MSH6** | ***E. coli* MutS** |
| --- | --- | --- |
| Insight | V398E | *N/A* |
| Insight | L449P | I53 |
| UMD | R482P | I92 |
| Insight | S514R | V111 |
| UMD | L585P | E169 |
| Insight | G686D | L240 |
| Insight | F706S | M260 |
| UMD | I745N | I279 |
| UMD | G770R | G303 |
| UMD | A861V | M368 |
| UMD | Y969C | V465 |
| Insight | L1063R | L558 |
| UMD | I1115K | L601 |
| UMD | C1129R | M609 |
| UMD | G1134V | G614 |
| UMD | G1139D | G619 |
| UMD | R1334Q | Q807 |

https://www.nigms.nih.gov/Pages/default.aspx
