# Supplementary figures and images for: Long-Range Signaling in MutS and MSH Homologs via Switching of Dynamic Communication Pathways
Source: PLoS Comput Biol. 2016 Oct 21;12(10):e1005159. doi: 10.1371/journal.pcbi.1005159 (PMC5074593; doi:10.1371/journal.pcbi.1005159)

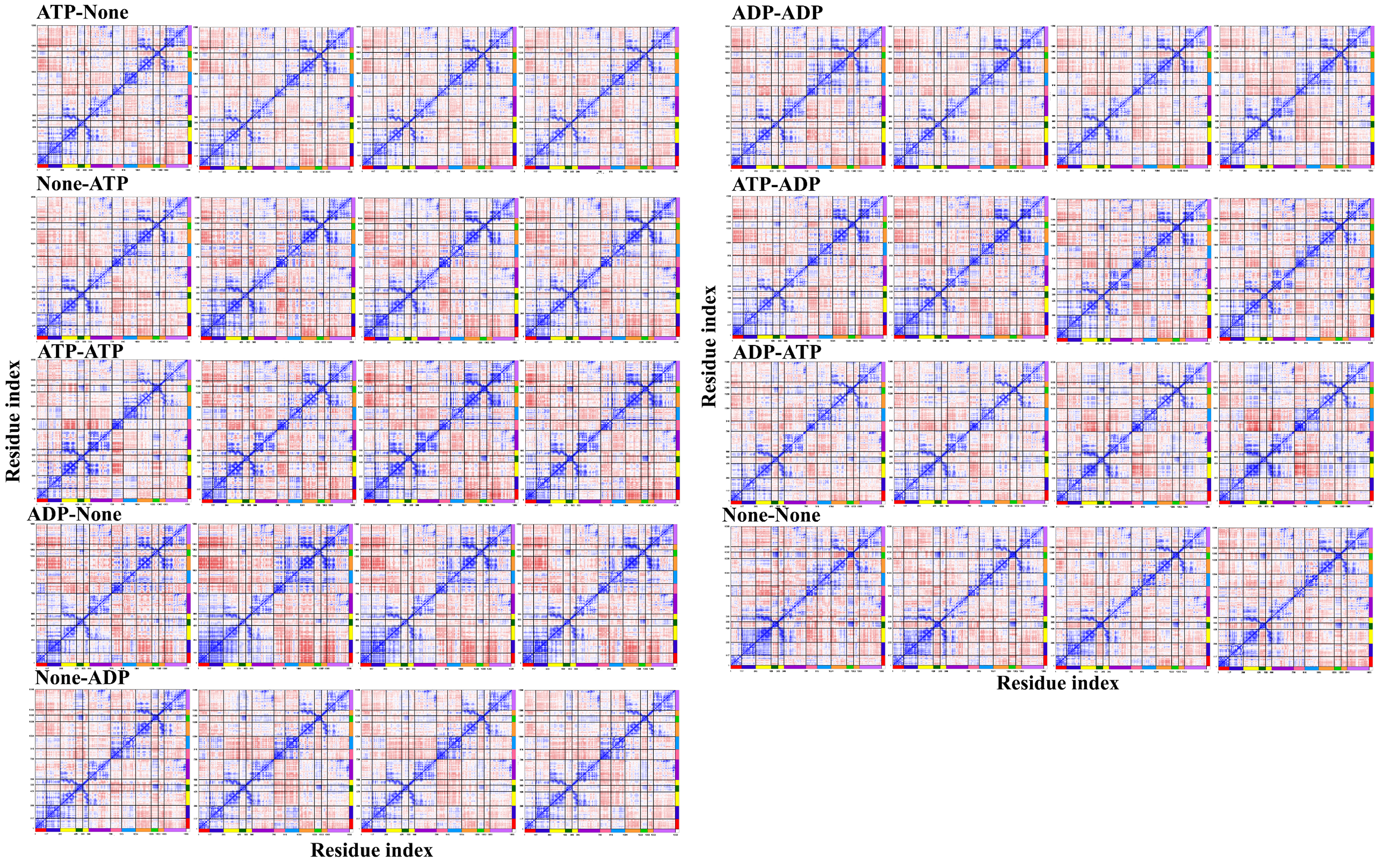

Supplement: S1 Fig — Dynamical cross-correlation matrices for Ca atoms for E. coli MutS simulated systems as a functions of nucleotides bound to ATPase domains using 50, 100, 150, and 200 ns of the simulation trajectories (from left to right). (TIF) [file pcbi.1005159.s006.tif]

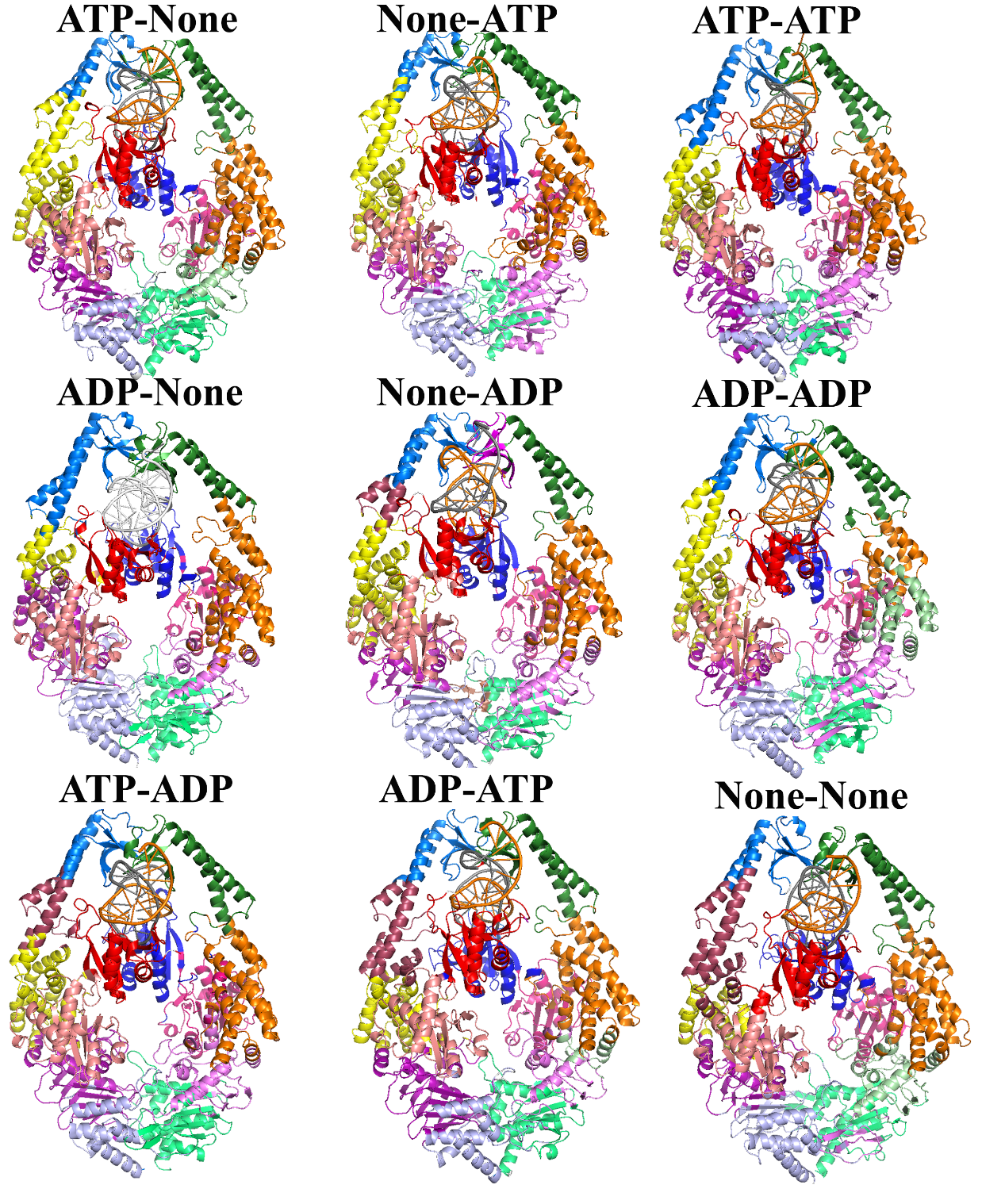

Supplement: S2 Fig — Communities of the MutS protein in all MutS systems (left: A monomer, right: B monomer). Different communities were colored differently. (TIF) [file pcbi.1005159.s007.tif]

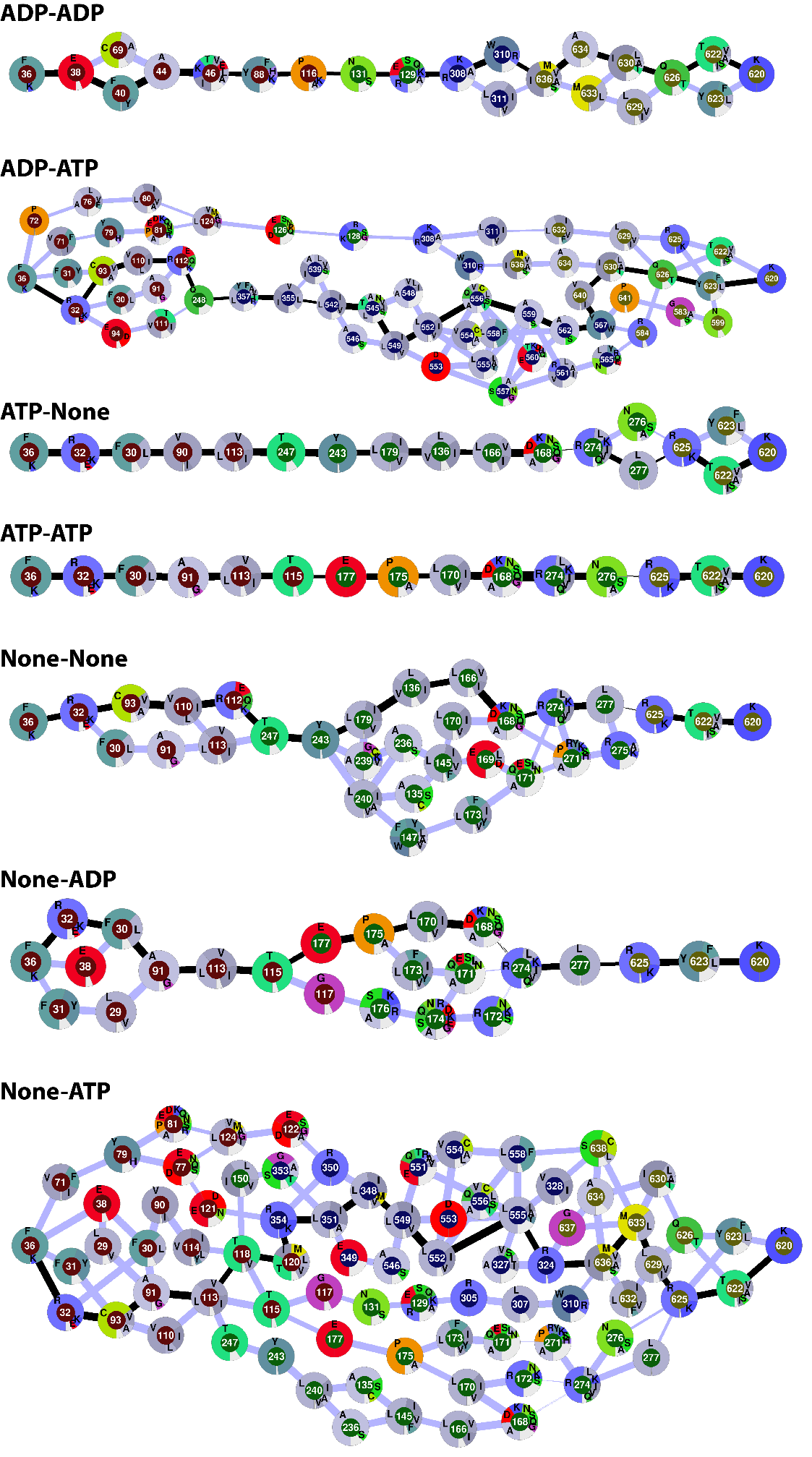

Supplement: S3 Fig — MBD-ATPaseA pathways for additional NTP states. (TIF) [file pcbi.1005159.s008.tif]

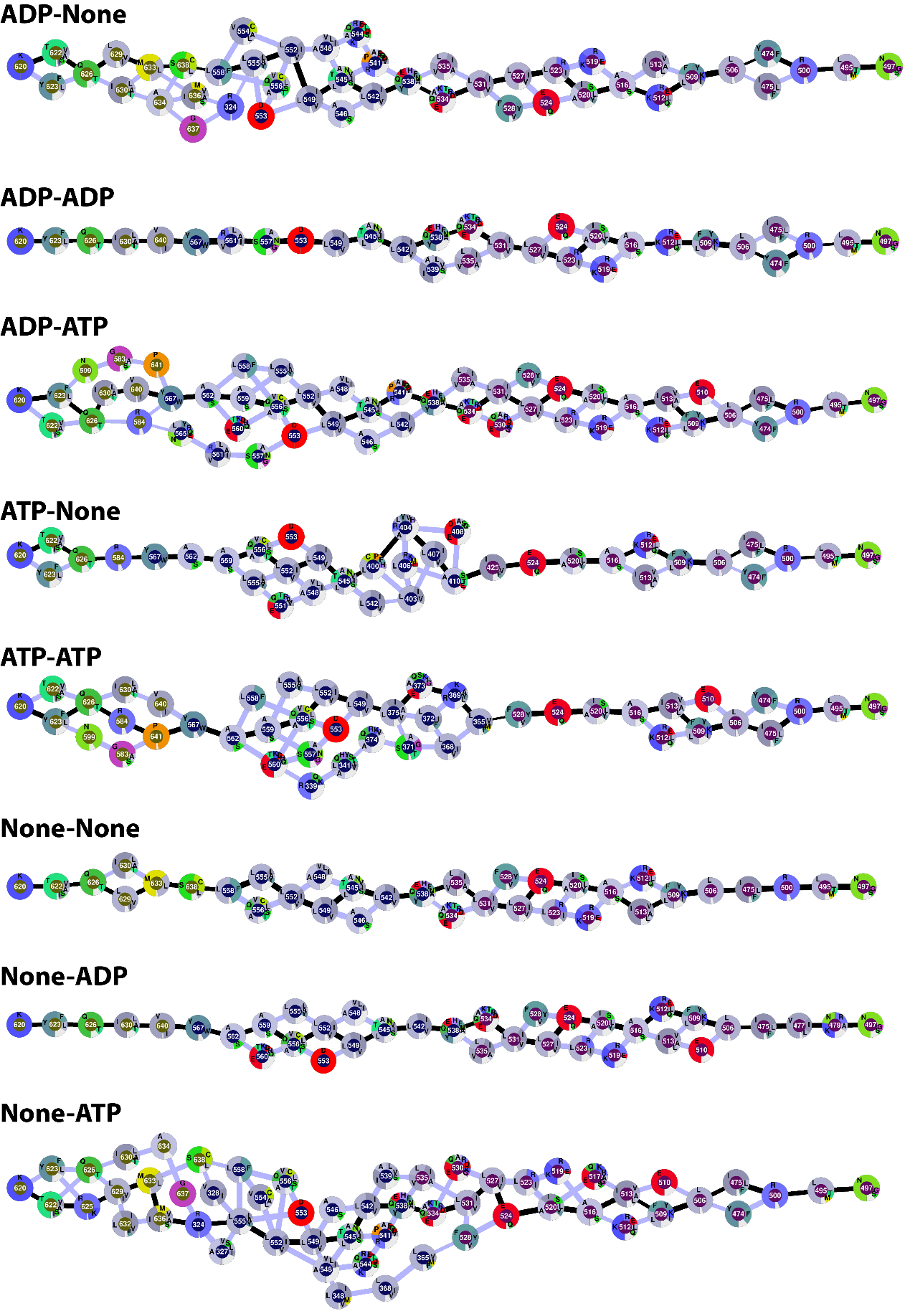

Supplement: S4 Fig — ATPaseA–clamp pathways for additional NTP states. (TIF) [file pcbi.1005159.s009.tif]

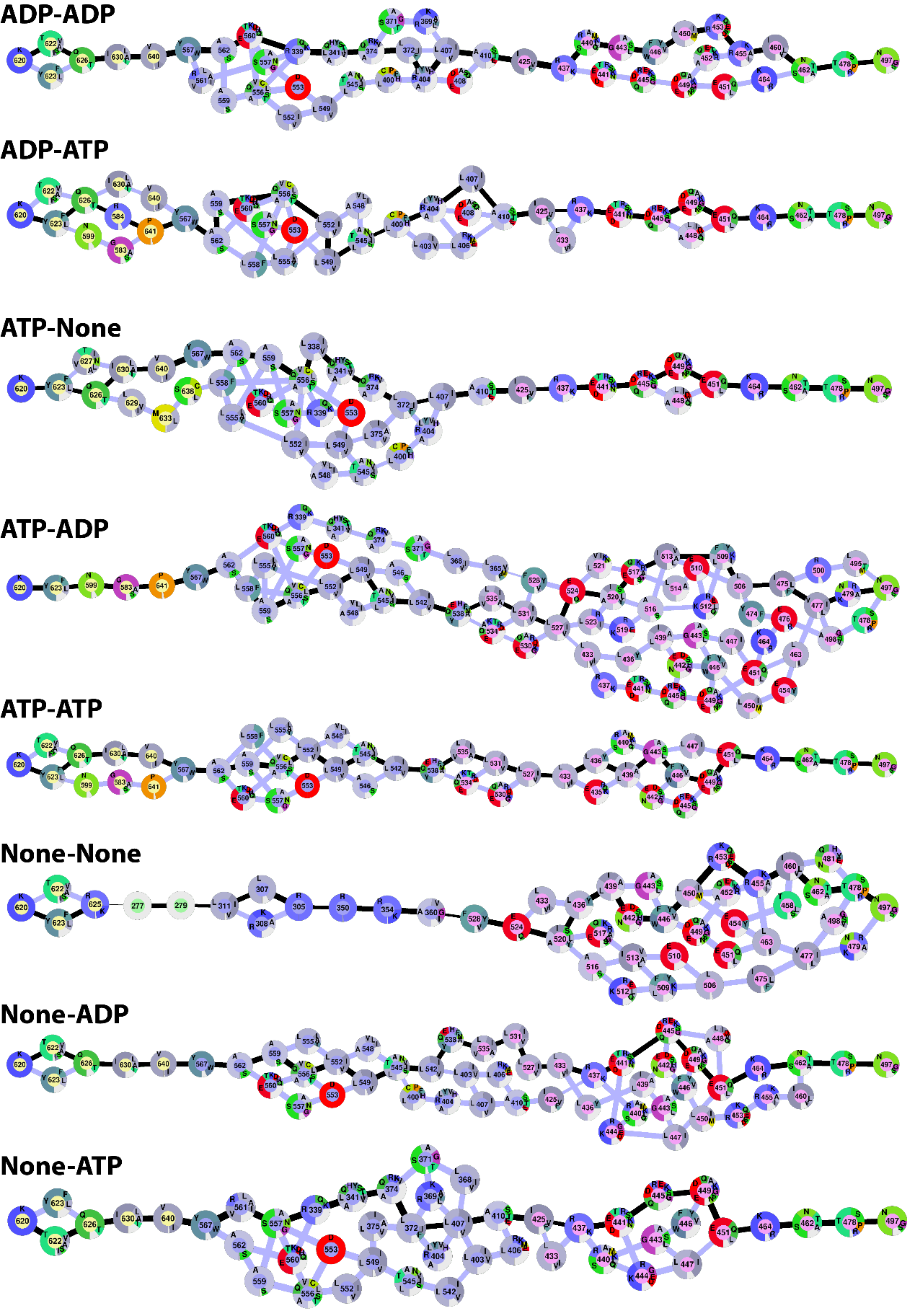

Supplement: S5 Fig — ATPaseB–clamp pathways for additional NTP states. (TIF) [file pcbi.1005159.s010.tif]

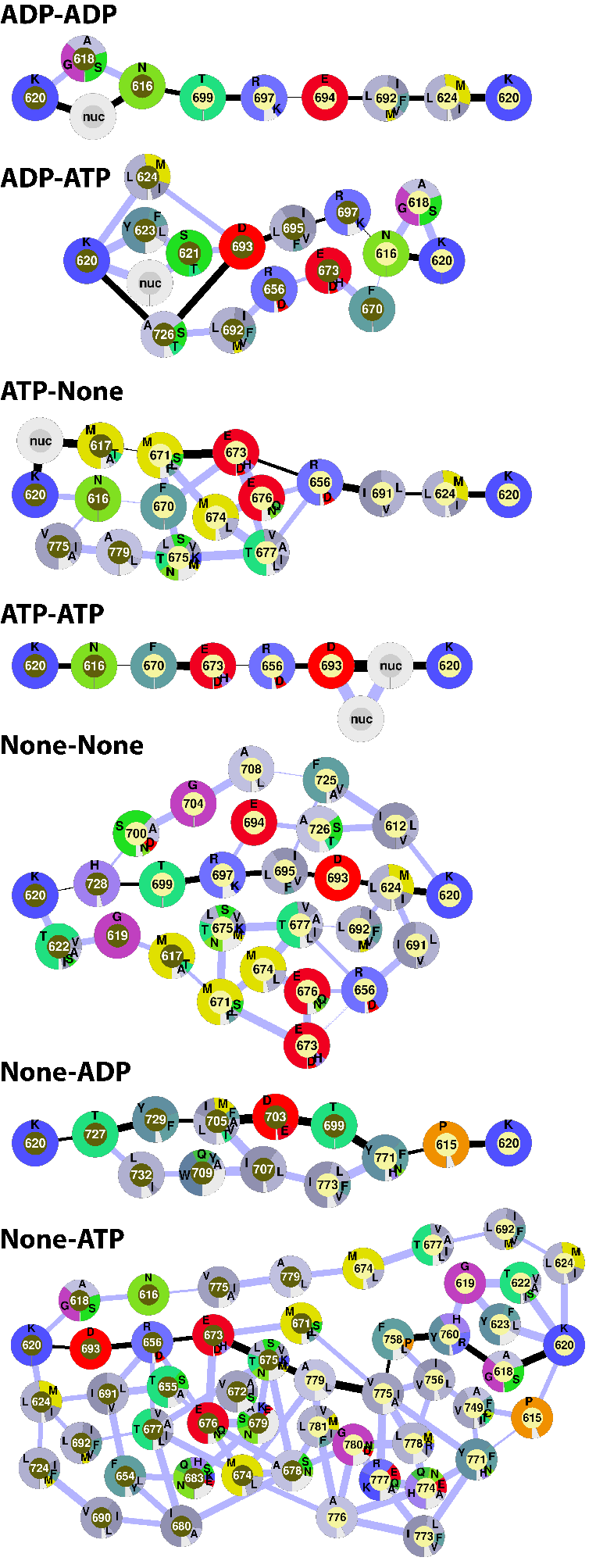

Supplement: S6 Fig — ATPaseA -ATPaseB pathways for additional NTP states. (TIF) [file pcbi.1005159.s011.tif]

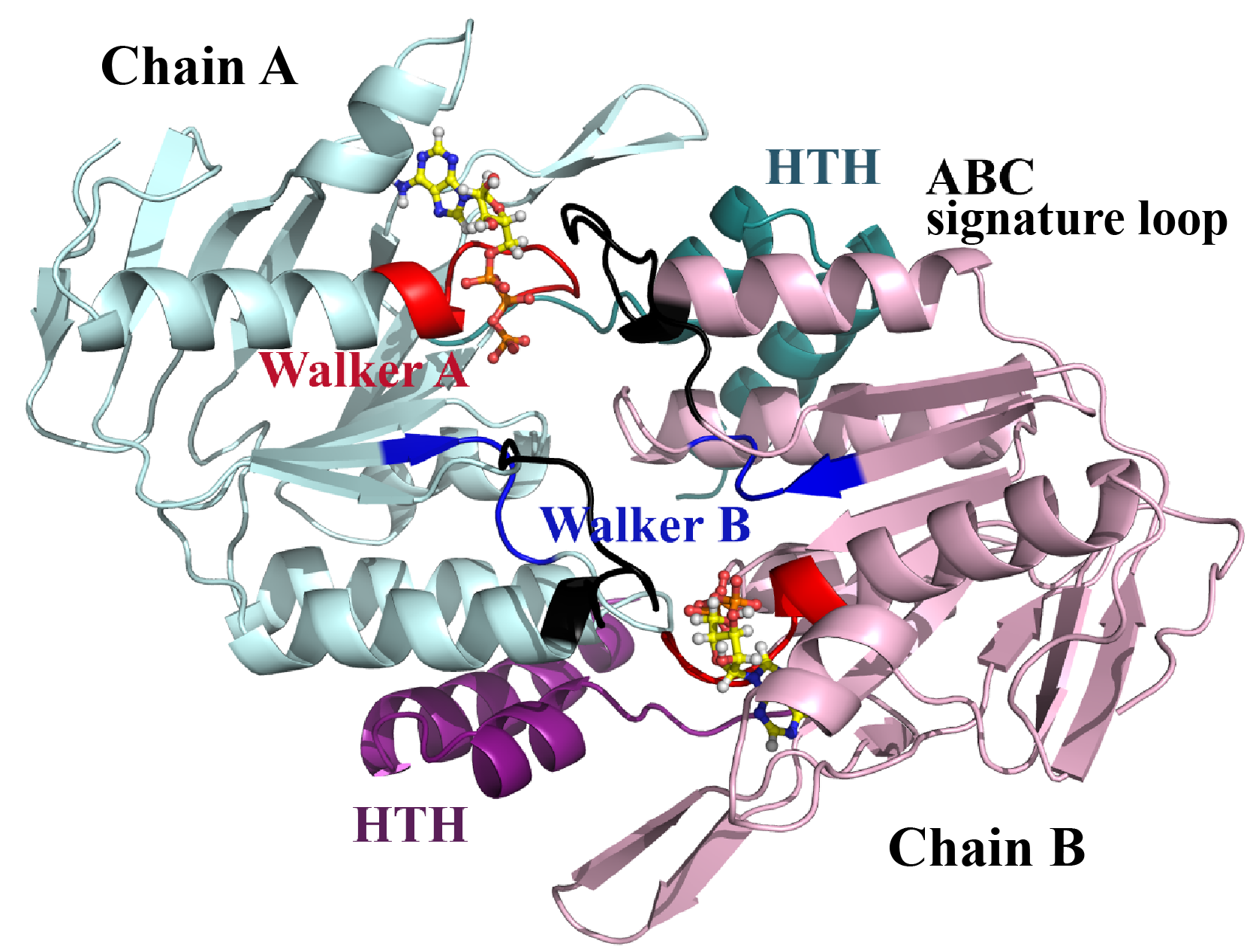

Supplement: S7 Fig — The conserved motifs in NBD domain. (TIF) [file pcbi.1005159.s012.tif]

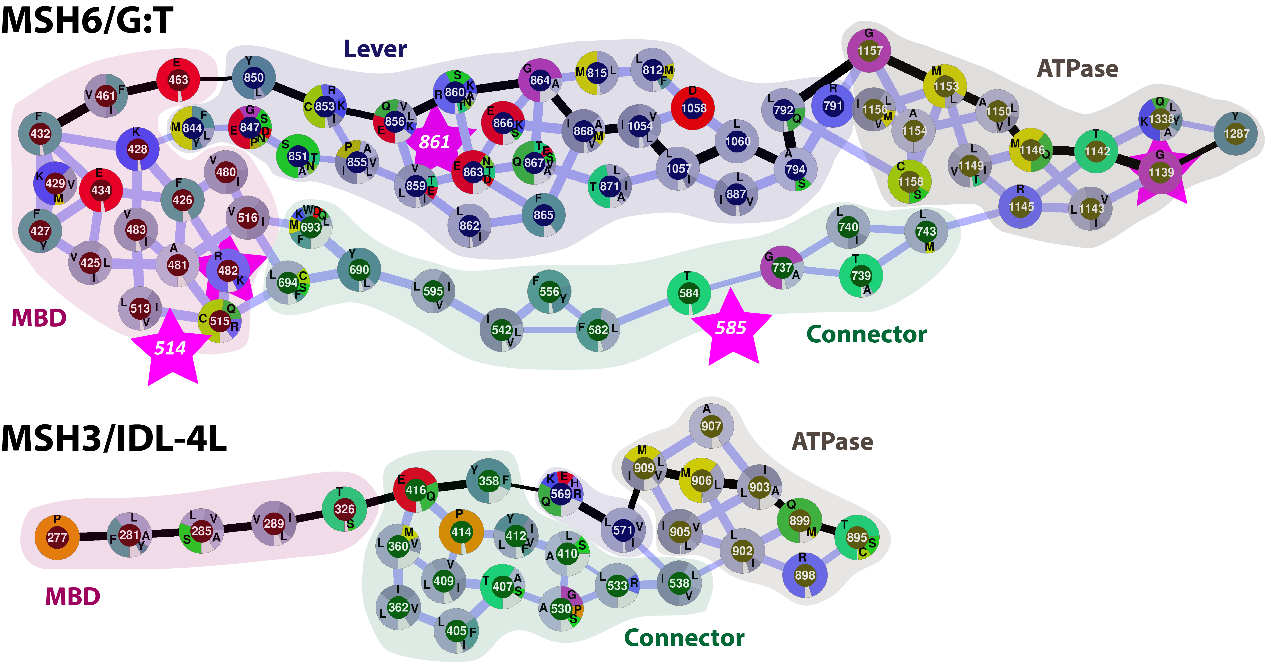

Supplement: S8 Fig — MBD-ATPase pathways in MutSα and MutSβ with native DNA substrates as in Fig 3. (TIF) [file pcbi.1005159.s013.tif]

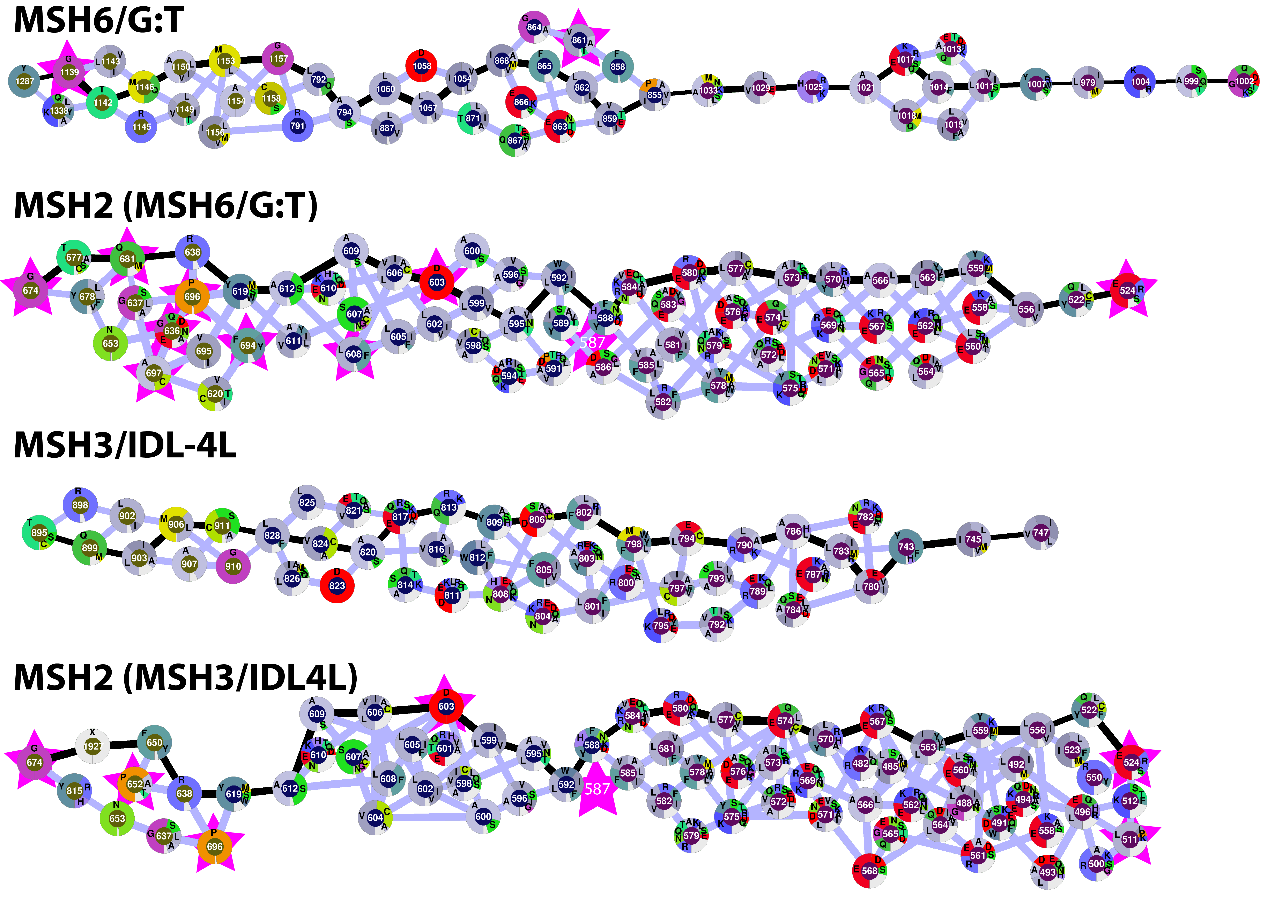

Supplement: S9 Fig — ATPase-clamp pathways in MutSα and MutSβ with native DNA substrates as in Fig 3. Cancer-associated mutations are highlighted with stars. (TIF) [file pcbi.1005159.s014.tif]
